# Efficient utilization of aerobic metabolism helps Tibetan locusts conquer hypoxia

**DOI:** 10.1186/1471-2164-14-631

**Published:** 2013-09-18

**Authors:** Dejian Zhao, Zhenyu Zhang, Arianne Cease, Jon Harrison, Le Kang

**Affiliations:** 1 State Key Laboratory of Integrated Management of Pest Insects and Rodents, Institute of Zoology, Chinese Academy of Sciences, Beijing, China; 2 School of Life Sciences, Arizona State University, Tempe, AZ, USA

**Keywords:** Hypoxia, Microarray, *Locusta migratoria*, Tibet

## Abstract

**Background:**

Responses to hypoxia have been investigated in many species; however, comparative studies between conspecific geographical populations at different altitudes are rare, especially for invertebrates. The migratory locust, *Locusta migratoria*, is widely distributed around the world, including on the high-altitude Tibetan Plateau (TP) and the low-altitude North China Plain (NP). TP locusts have inhabited Tibetan Plateau for over 34,000 years and thus probably have evolved superior capacity to cope with hypoxia.

**Results:**

Here we compared the hypoxic responses of TP and NP locusts from morphological, behavioral, and physiological perspectives. We found that TP locusts were more tolerant of extreme hypoxia than NP locusts. To evaluate why TP locusts respond to extreme hypoxia differently from NP locusts, we subjected them to extreme hypoxia and compared their transcriptional responses. We found that the aerobic metabolism was less affected in TP locusts than in NP locusts. RNAi disruption of PDHE1β, an entry gene from glycolysis to TCA cycle, increased the ratio of stupor in TP locusts and decreased the ATP content of TP locusts in hypoxia, confirming that aerobic metabolism is critical for TP locusts to maintain activity in hypoxia.

**Conclusions:**

Our results indicate that TP and NP locusts have undergone divergence in hypoxia tolerance. These findings also indicate that insects can adapt to hypoxic pressure by modulating basic metabolic processes.

## Background

Animals with wide altitudinal distribution are subjected to different ambient oxygen levels, representing a promising system for disclosing the mechanism of hypoxia adaptation. These animals have been effectively used to elucidate the molecular and physiological foundations of hypoxia adaptation. For example, the bar-headed geese, *Anser indicus*, that can fly at 9 km elevation during migration, possesses a nonsynonymous mutation of COX3 gene (Trp116→Arg) leading to a higher COX affinity and COX activity as compared to low-altitude geese [1]. The deer mouse, *Peromyscus maniculatus*, is continuously distributed from sea-level elevation to high altitude above 4,300 m. DNA sequences of two α-globin genes revealed that Asp64→Gly substitution appeared in high-altitude populations of *P. maniculatus* at unusually high frequency and could substantially increase oxygen-binding affinity of α-globin [2]. Several hypoxia-responsive genes, including EPAS1, EGLN1 and PPARA, were under positive selection in Tibetan natives compared to lowlanders [3-5]. Although this approach holds much promise, currently it is mainly applied to vertebrates rather than invertebrates.

The migratory locust, *Locusta migratoria*, is the most widely distributed locust species in the world. Despite of their wide geographic distribution, the migratory locust around the world is taxonomically the same species [6]. A recent research revealed that the locust population of *L. migratoria* in the world could be divided into two distinct lineages, the Northern lineage and the Southern lineage, both of which were originated from Africa and then separated 895,000 years ago [6]. TP locusts, previously identified as *L. migratoria tibetensis*[7], belong to the Southern lineage, whereas NP locusts belong to the Northern lineage. Multilocus microsatellite genotyping analysis revealed that TP locusts are genetically distinct from NP locusts and they have experienced adaptive differentiation coupled to Quaternary glaciations events [8]. A Bayesian phylogram based on locust mitochondrial sequences also supported that TP locusts had no frequent genetic exchange with NP locusts [6]. TP locusts likely expanded into Tibet 34,000 and 40,000 years ago and have since inhabited the Tibetan Plateau [6]. Although the partial oxygen pressure (*p*O_2_) in Tibet (~13 kPa) is above the critical oxygen pressure of locusts (~3 kPa) [9,10], the Tibetan *p*O_2_ still exerts selective pressures on locusts [11]. Long-term habitation to the Tibetan Plateau probably has adapted TP locusts to the chronic low-oxygen atmosphere, conferring on them superior tolerance of hypoxia.

The evolution is ultimately attributed to genetic mutations which may occur in the coding region or the regulatory region of a gene. If mutations occur in the coding region, they may modify the molecular function of the gene; if in the regulatory region, they may alter its gene expression levels. The genetic variations in the coding regions are successfully evaluated in Tibetans and lowlanders by using human SNP arrays [3,4] and human exon capture arrays [5]. However, such arrays are not available for locusts. Instead, we have developed the locust microarray platform, which has been proved to be an effective approach to study locust gene expression profiles [12-14]. Comparison of gene expression patterns using locust microarrays is an alternate approach to understand the natural selection and adaptive evolution of TP locusts [15,16].

In this study, we confirmed the superior capacity of TP locusts to tolerate hypoxia through a series of phenotypic assays. Then we examined gene expression profiles of field and laboratory locusts using locust microarrays and found that TP locusts utilized the aerobic metabolism in a better way than NP locusts, which was further confirmed by RNAi experiments. Our data demonstrate that TP and NP locusts have diverged in hypoxia tolerance by different modulation of basic metabolism.

## Results

### Phenotypic differences between NP and TP locusts

**Figure 1:**
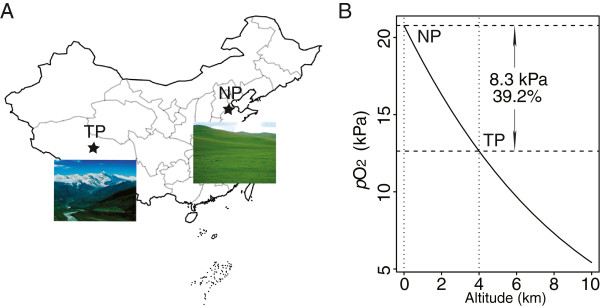
**Natural living condition of sampled locusts. (A)** Sample locations of the migratory locust. Tibetan population was located on Tibetan Plateau (TP) with average altitude over 4 km, and North China population on North China Plain (NP) with average altitude below 50 m. **(B)** Partial oxygen pressures (*p*O_2_) of sample locations. The formula for atmosphere pressure in terms of altitude was given in the CRC Handbook of Chemistry and Physics (1996 edition) and was modified to calculate *p*O_2_. The *p*O2 was calculated using the formula $\text{pO}2=0.0020946\times {\left(\frac{44331.514-Z}{11880.516}\right)}^{5.255877}$, where *p*O_2_ is pressure in kilopascals and Z is altitude in meters.

**Figure 2:**
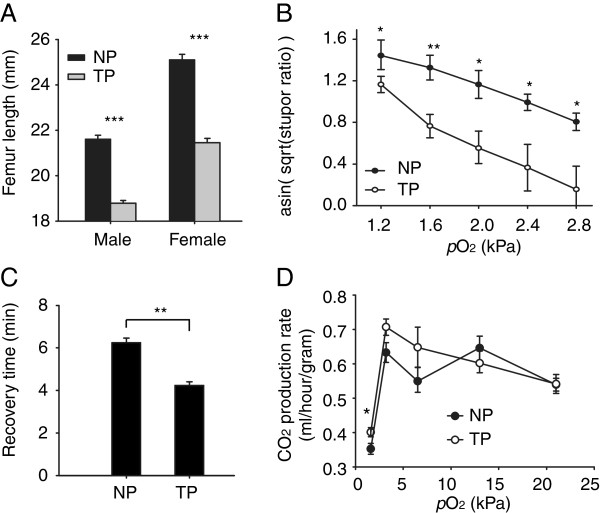
**Phenotypic differences between NP and TP locusts. (A)** Femur length. Data was represented as Mean ± SD (Student’s *t*-test, ***p < 0.001, n = 30). **(B)** Stupor ratios of locusts under different levels of partial oxygen pressure (*p*O_2_). Square roots of stupor ratios were calculated and then were arcsine-transformed to conduct Student’s *t*-test. Data were presented as Mean ± SD (Student’s *t*-test,*p < 0.05, **p < 0.01, n = 6). **(C)** Recovery time. Data were presented as Mean ± SD (Student’s *t*-test, **p <0.01, n = 30). **(D)** CO_2_ production rate of locusts under a series of *p*O_2_, or 1.6 kPa, 3.2 kPa, 6.5 kPa, 13 kPa and 21 kPa. Data were presented as Mean ± SD (Student’s *t*-test, *p < 0.05, n = 36).

The migratory locust *Locusta migratoria* is widely distributed in China, both on the low-altitude North China Plain (NP) and on the high-altitude Tibetan Plateau (TP) (Figure 1A). The natural habitats of NP and TP locusts show great differences, one of which is the partial oxygen pressure (*p*O_2_) which decreases around 40% in Tibet compared to that in North China (Figure 1B). To test whether NP and TP locusts have evolved different sensitivity to hypoxia, we compared their phenotypic traits from morphological, behavioral and physiological perspectives. First, we compared their body size by using femur length as an index. The average femur length of male and female NP locusts was 21.59 mm and 25.21 mm; 13.7% and 17% larger than that of male and female TP locusts, respectively (Figure 2A). Second, to directly compare the capacity of NP and TP locusts to tolerate hypoxia, we measured the ratio of locusts exhibiting stupor by subjecting them to five different levels of extreme hypoxia. When *p*O_2_ decreased from 2.8 kPa to 1.2 kPa, the stupor ratio of NP locusts rose from 52% to 96% while that of TP locusts increased from 6% to 84%. Under all the tested oxygen levels, the stupor ratios of TP locusts were smaller than those of NP locusts, with the biggest difference at 1.6 kPa *p*O_2_ (Figure 2B). Third, to evaluate the effects of hypoxic injury on TP and NP locusts, we compared the time required to recover from stupor. It took TP locusts 4.2 min to recover from stupor, significantly less than 6.2 min required by NP locusts (Figure 2C). Finally, to evaluate the effect of hypoxia on aerobic metabolism, we compared metabolic rates under a series of *p*O_2_ (1.6 kPa, 3.2 kPa, 6.5 kPa, 13 kPa and 21 kPa) by measuring their CO_2_ production rates. Locusts did not show significant differences in metabolic rates when the *p*O_2_ was equal to or above 3.2 kPa. When *p*O_2_ reached 1.6 kPa, CO_2_ production rates of both populations plummeted drastically; however, the TP locusts still showed higher CO_2_ production rates than the NP locusts (Figure 2D). Taken together, these phenotypic differences suggest that TP locusts have evolved a better capacity to cope with hypoxia than NP locusts.

### Gene expression profiles of field NP and TP locusts

**Figure 3:**
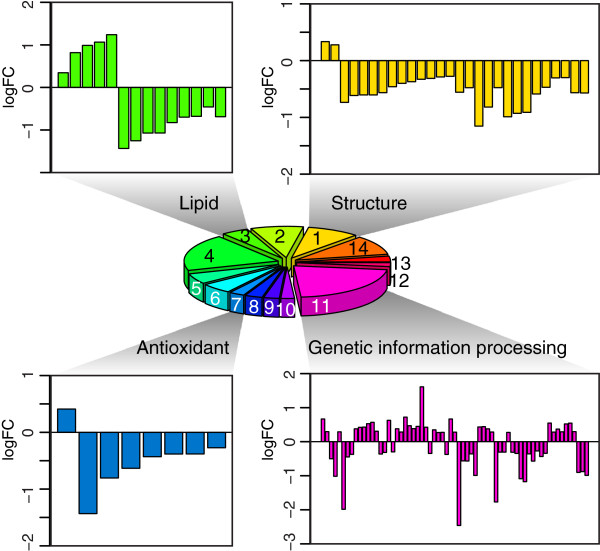
**Comparison of gene expression between the field population of NP and TP locusts.** The differentially expressed genes (DEGs) were classified into 14 functional categories as follows: 1, structural genes; 2, immunity and detoxification; 3, lipid metabolism; 4, collection of various functions; 5, neuronal development and regulation; 6, growth and development; 7, antioxidant; 8, enzymes and inhibitors; 9, transport; 10, amino acid metabolism; 11, genetic information processing; 12, cell cycle and apoptosis; 13, reproduction; 14, carbohydrate metabolism. See Additional file 1 for details in each category.

To explore how NP and TP locusts diverged at the transcriptional level after adapting to different habitats, we compared the gene expression of thoracic muscles between the field populations. We identified 324 differentially expressed genes (DEGs) up-regulated in TP locusts and 555 DEGs up-regulated in NP locusts (Additional file 1). Of the 879 genes, only 24 were among the 235 previously identified target genes of hypoxia-inducible factor [11]. Based on GO, KEGG and NCBI annotations, DEGs were further classified into 14 functional categories (Figure 3, Additional file 1), which include a variety of basic functions, e.g. cellular structure, lipid metabolism, antioxidant processes, DNA repair and gene expression. In the field-caught TP locusts, structural genes involved in the assembly of cytoskeleton, muscles and cuticle were suppressed (Figure 3; Category 1, Additional file 1), such as tubulin (LM04131, LM00737, LM03863, Additional file 1), tropomyosin (LM00621, Additional file 1) and endocuticle structural glycoprotein (LM00591, LM00412, Additional file 1). Genes related to fatty acid synthesis and β oxidation were also suppressed in TP locusts (Figure 3; Category 3, Additional file 1), such as acetyl-coenzyme A synthetase (LM06760 and LM06776, Additional file 1) which activates acetate so that it can be used for lipid synthesis [17] and delta(3,5)-delta(2,4)-dienoyl-CoA isomerase (LM01097, Additional file 1) which catalyzes β oxidation of unsaturated fatty acids [18]. Besides, the genes in the antioxidant systems were suppressed in TP locusts as well (Figure 3; Category 7, Additional file 1), e.g. superoxide dismutase (LM03935, Additional file 1), peroxiredoxin (LM00341, Additional file 1), and glutaredoxin (LM00278, Additional file 1). Genes involved in genetic information processing outnumbered any other categories (Figure 3; Category 11, Additional file 1). These genes were involved in DNA repair, transcription, translation, and protein degradation. Of particular interest, two genes involved in repairing UV-damaged DNA were up-regulated in TP locusts, namely ubiquitin-conjugating enzyme E2 (LM02929, Additional file 1) which is required for postreplication repair of UV-damaged DNA [19] and UV excision repair protein RAD23 homolog B (LM02985, Additional file 1) which is involved in global genome nucleotide excision repair by acting as component of the XPC complex [20]. In contrast, a gene involved in recombination repair was up-regulated in NP locusts (LM03194, Additional file 1), which promotes homologous recombination at sites of DNA damage [21]. The gene expression profiles showed that multiple factors contributed to the transcriptional differences between the field NP and TP locusts.

To understand how field TP locusts respond to the high-altitude environments, we identified the significantly enhanced pathways in field-collected TP locusts (Additional file 2). Most of the enhanced pathways involve amino acid metabolism such as glycine, serine and threonine metabolism. Of particular interest is the selenoamino acid metabolism, which may help reduce the oxidative stress [22] and decrease the radiation damage to amino acid and proteins [23]. Citrate cycle (TCA cycle) was also up-regulated, indicating that the aerobic pathway was possibly enhanced to produce sufficient energy molecules in field TP locusts. Interestingly, olfactory transduction was also strengthened, which is possibly related to food foraging and feeding in the field TP locusts.

### Gene expression profiles of laboratory NP and TP locusts under extreme hypoxia

**Figure 4:**
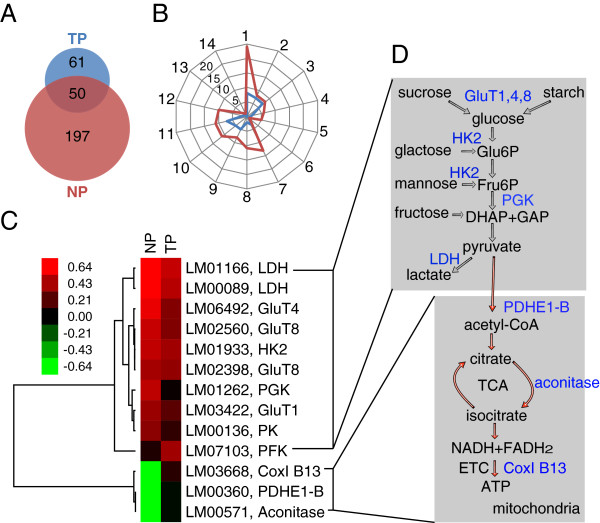
**Comparison of transcriptional responses to extreme hypoxia between the lab population of NP and TP locusts. (A)** Numbers of differentially expressed genes (DEGs) in NP and TP locusts exposed to extreme hypoxia. **(B)** Gene ontology (GO) classification of DEGs. The fourteen radii represent fourteen GO categories while the red and blue lines indicate the number of DEGs in each category. 1, GO:0044238 primary metabolic process; 2, GO:0051234 establishment of localization; 3, GO:0006810 transport; 4, GO:0050789 regulation of biological process; 5, GO:0007017 microtubule-based process; 6, GO:0008219 cell death; 7, GO:0005622 intracellular; 8, GO:0043226 organelle; 9, GO:0016020 membrane; 10, GO:0016491 oxidoreductase activity; 11, GO:0003676 nucleic acid binding; 12, GO:0042302 structural constituent of cuticle; 13, GO:0003700 transcription factor activity; 14, GO:0004601 peroxidase activity. **(C)** Cluster analysis of DEGs involved in glycolysis and energy production processes. Hierarchical clusters were calculated using Euclidean distance similarity metric and average linkage method. **(D)** Diagram of functional relationship of clustered genes. The upper part of the diagram represents several anaerobic metabolic processes, mainly glycolysis; the lower part represents several aerobic metabolic processes which occur mainly in mitochondria.

NP and TP locusts responded differently to extreme hypoxia (Figure 2B-D). To understand what contributed to the superior tolerance of extreme hypoxia for TP locusts, we compared the transcriptional responses of their thoracic muscles to extreme hypoxia (1.6 kPa *p*O_2_). We identified 247 hypoxia-responsive genes from NP locusts and only 111 from TP locusts (Figure 4A, Additional files 3 and 4). The expression levels of 10 genes, including both DEGs and non-DEGs, were confirmed by real-time quantitative PCR (RT-qPCR) (Additional files 5 and 6). The genes were classified into 14 gene ontology (GO) categories. Metabolic processes had the largest number of DEGs and the biggest difference in DEGs number between the NP and TP locusts (Figure 4B). Further investigation of the DEGs in metabolic processes showed that these genes were mainly involved in glycolysis, the citrate cycle, and the electron transfer chain (Figure 4C, D). Genes involved in anaerobic metabolism were up-regulated in both NP and TP locusts (Figure 4C, D), including lactate dehydrogenase (LDH), glucose transporter (GluT), hexokinase type 2 (HK2), phosphoglycerate kinase (PGK), pyruvate kinase (PK), and 6-phosphofructo-2-kinase/fructose-2,6-bisphosphatase 4 (PFK). In contrast, genes involved in aerobic processes were down-regulated only in NP locusts, including complex I subunit B13, pyruvate dehydrogenase (PDH) E1 component subunit beta and aconitase (Figure 4C).

### Role of aerobic metabolism in the tolerance of extreme hypoxia

**Figure 5:**
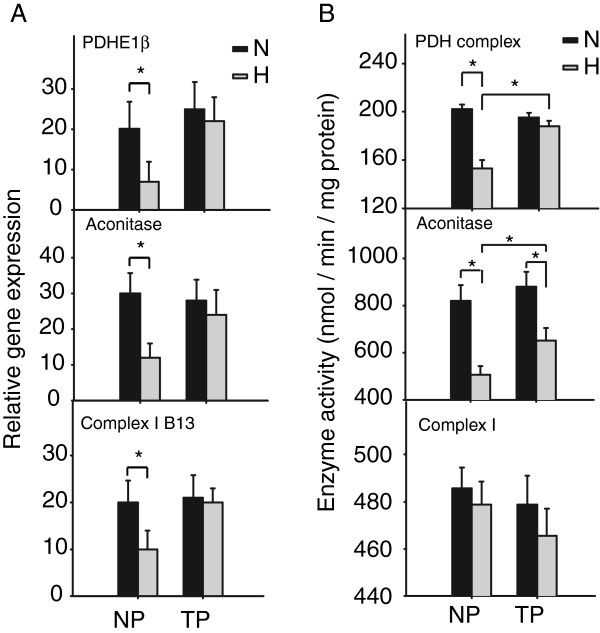
**Confirmation of differences in aerobic metabolic processes between NP and TP locusts under extreme hypoxia. (A)** Quantification of gene expression of three DEGs in aerobic metabolic processes. Data were presented as Mean ± SD (Student’s *t*-test, *p < 0.05, n = 6). N: normoxia; H: hypoxia. **(B)** Enzyme activities in aerobic metabolic processes. Data were presented as Mean ± SD (Student’s *t*-test, *p < 0.05, n = 6). N: normoxia; H: hypoxia.

Based on the facts that TP locusts showed higher aerobic respiration rates than the NP locusts under extreme hypoxia (Figure 2D) and the aerobic metabolic processes were not as severely suppressed in TP locusts as in NP locusts (Figure 4C-D), we speculate that the aerobic metabolism plays an important role in the adaptation of TP locusts to extreme hypoxia. We first examined the gene expression levels of three DEGs under extreme hypoxia: PDH E1 component subunit beta, aconitase, and complex I subunit B13. Consistent with microarray results, the three genes were all suppressed in NP locusts and not significantly affected in TP locusts (Figure 5A). We then measured the enzymatic activities of PDH complex, aconitase, and complex I. We found in NP locusts that enzymatic activities of PDH complex and aconitase were reduced under extreme hypoxia while that of complex I were not significantly altered (Figure 5B). In TP locusts, the activity of aconitase was also reduced, but it remained higher than that in NP locusts; similarly, the activity of PDH complex was also higher in TP locusts than in NP locusts under extreme hypoxia (Figure 5B).

**Figure 6:**
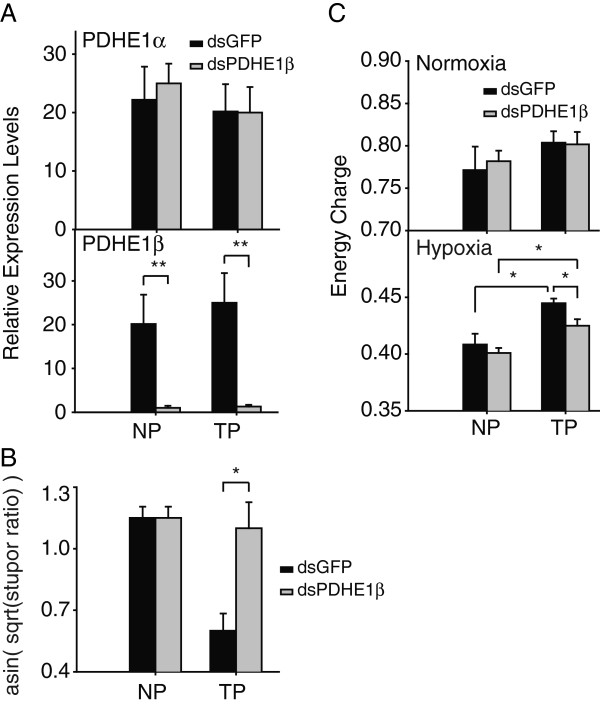
**Effects of disrupting PDHE1β with RNAi on NP and TP locusts. (A)** Gene expression of PDHE1α and PDHE1β after RNAi. Data were presented as Mean ± SD (Student’s *t*-test,*p < 0.05, n = 6). **(B)** Stupor ratios of NP and TP locusts under 2.0 kPa *p*O_2_ after RNAi. Square roots of stupor ratios were calculated and then were arcsine-transformed to conduct Student’s *t*-test. Data were presented as Mean ± SD (Student’s *t*-test,*p < 0.05, n = 6). **(C)** Energy charge (EC) in flight muscles of NP and TP locusts after RNAi. $\text{EC}=\frac{\left[\text{ATP}\right]+0.5\times \left[\text{ADP}\right]}{\left[\text{ATP}\right]+\left[\text{ADP}\right]+\left[\text{AMP}\right]}$, [ATP], [ADP] and [AMP] represent the concentrations of ATP, ADP and AMP, respectively. Data were presented as Mean ± SD (Student’s *t*-test,*p < 0.05, n = 6).

To further confirm our speculation, we blocked the aerobic metabolic branch by disrupting PDHE1β using RNAi [24], an entry gene from glycolysis to TCA cycle (Figure 4D). The expression level of PDHE1β was drastically reduced by 20-fold while expression of PDHE1α, partner of PDHE1β, was not significantly affected in both TP and NP locusts (Figure 6A). A behavioral test under hypoxia showed that the stupor ratio of TP locusts with PDHE1β disrupted was 33.3% higher than that in control locusts (Figure 6B). To further evaluate the effect of RNAi, we measured the contents of AMP, ADP, and ATP, and calculated the energy charge (EC), which is an indicator of cellular energy status. The contents of these molecules did not change significantly in both TP and NP locusts under normoxia after RNAi (Additional file 7A-C). However, when locusts were placed under extreme hypoxia (1.6 kPa *p*O_2_), the contents of AMP, ADP, and ATP changed significantly in TP locusts after RNAi compared to control (dsGFP). When PDHE1β was disrupted in TP, AMP was 1.7 nmol/mg higher than control, ADP 2.6 nmol/mg lower, and ATP 1.9 nmol/mg higher (Additional file 7D-F). Under normoxia, RNAi did not alter EC significantly after disrupting PDHE1β (Figure 6C). However, under hypoxia, EC values of NP and TP locusts decreased in both control and treated locusts; TP locusts with PDHE1β disrupted had lower EC values than the control (Figure 6C). Taken together, gene expression, enzymatic activities, and RNAi data support that aerobic metabolism plays an important role in the adaptation of TP locusts to extreme hypoxia.

To investigate whether the gene PDHE1β undergoes genetic variations between TP and NP locusts, we sequenced the coding region of PDHE1β in 15 individuals of TP and NP locusts (Additional file 5). Multiple sequence alignment revealed no significant genetic variation in the coding region of PDHE1β (Additional file 8).

## Discussion

The wide distribution of *L. migratoria* makes it possible that locusts from different habitats may have different adaptations to specific environments. TP locusts likely expanded into Tibet 34,000 and 40,000 years ago [6] and have since inhabited the Tibetan Plateau. Long-term habitation to the Tibetan Plateau probably has adapted TP locusts to the chronic low-oxygen atmosphere, conferring on them superior tolerance of hypoxia.

We examined four phenotypic traits in TP and NP locusts (Figure 2), all of which demonstrate that TP locusts are better prepared for hypoxia tolerance. Smaller body size in TP locusts is possibly a response to the chronic hypoxia as noted in fruit flies [25,26]. Using hypoxic stupor - a common response to extreme hypoxia in insects [27] - as criteria, we found that TP locusts were more tolerant of extreme hypoxia. Recovery time from stupor, once used as a criteria to screen for hypoxia-sensitive fruit flies [28], revealed that NP locusts were sensitive to hypoxic injury, possibly related to longer polysynaptic transmission in central nervous system [28]. The respiration rates under a series of hypoxia suggested that hypoxia suppressed aerobic respiration drastically when *p*O_2_ was near the critical value as reported in American grasshopper, *Schistocerca americana*[10]; however, TP locusts maintained higher respiration rates than NP locusts, indicating TP locusts were able to utilize oxygen more efficiently under extreme hypoxia. Taken together, these results indicate that the two geographical populations have different thresholds for hypoxia tolerance.

The gene expression profiles of the field-collected NP and TP locusts showed that multiple factors contributed to their transcriptional differences (Figure 3). One major difference between the field habitats of NP and TP locusts is the oxygen content, which decreases by 39.2% in Tibet compared to in North China Plain. The HIF target genes and antioxidant genes were differentially expressed between field NP and TP locusts, possibly due to the different oxygen content. The antioxidant genes were suppressed in TP locusts, which is possibly due to a decreased need to avoid oxygen toxicity [29]. Another important difference is the UV radiation, which is more severe in Tibet. UV radiation can induce a variety of mutagenic and cytotoxic DNA lesions, which are harmful to the genetic stability [30]. The up-regulation of genes repairing UV-damaged DNA reflects the different UV radiation in the field. In addition, other factors such as temperature and nutrition can also affect the lipid metabolism and cell growth [31,32]. To identify the genes that contributed to the enhanced tolerance of hypoxia in TP locusts, we took measures to minimize the effects of factors other than oxygen.

TP locusts showed better tolerance of extreme hypoxia than NP locusts (Figure 2) and the capacity of TP locusts to modulate basic metabolism contributed to this superiority (Figures 4 and 5). Altering the metabolic pathways to produce more ATP is a common strategy to cope with hypoxia for living organisms. For example, metabolomic and computational analyses of fruitfly flight muscles under 0.5% oxygen levels showed that conversion of pyruvate from lactate to alanine and acetate could convey hypoxia tolerance by improving ATP-producing efficiency per glucose [33]. Flux-balance analyses showed that flies adapted to 4% oxygen levels produced more ATP per glucose by lowering pyruvate carboxylase flux and preferring the usage of Complex I to Complex II [26,34].

The metabolic processes of TCA and ETC. were less suppressed in TP locusts (Figures 4 and 5). These results indicate that TP locusts could better utilize aerobic metabolism than NP locusts to produce ATP under extreme hypoxia, and is consistent with the fact that TP locusts had a higher respiration rate under extreme hypoxia (Figure 2D). It appears that higher efficiency to utilize limited oxygen to produce more ATP helps TP locusts conquer the extreme hypoxia. As observed in mammals, the COX subunit was switched from COX4-1 to COX4-2 to maintain optimal efficiency of mitochondrial respiration under 1% oxygen levels [35]. We confirmed the importance of aerobic metabolism in the tolerance of extreme hypoxia for TP locusts through RNA interference (Figure 6). Our results suggest that disrupting PDHE1β, a subunit of E1 component in pyruvate dehydrogenase complex (PDH), dramatically suppressed energy charge and increased stupor in hypoxia for TP locusts, indicating aerobic metabolism was critical for TP locusts to resist extreme hypoxia. PDH catalyzes an irreversible reaction that links glycolysis in the cytoplasm and TCA cycle in the mitochondrion, controlling the entry from anaerobic metabolism to aerobic metabolism [36]. Disrupting PDHE1β would prevent transfer of substrates through flip-flop action [37], and thus prohibit pyruvate from entry into the TCA cycle, in which NADH and FADH_2_ are produced for following production of ATP. Missense mutations in PDHE1α gene, which weaken the transfer of substrates as we did using RNAi, lead to PDH deficiency and are associated with lactic acidosis and central nervous system dysfunction [36,38]. This suggests that decreased expression of PDHE1β in NP locusts under hypoxia probably contributes to their vulnerability to extreme hypoxia and unaffected expression of PDHE1β help TP locusts conquer extreme hypoxia.

TP locusts adapted to altitude hypoxia and evolved superior capacity to produce ATP efficiently through aerobic metabolism under hypoxia. The locust strategy is well consistent with that taken by vertebrates. For example, genetic comparison between Tibetan natives and lowlanders indicated that one SNP in EPAS1 gene showed significant difference in frequency among the two populations, which is associated with erythrocyte abundance and hemoglobin concentration that is proportional to oxygen transport [4,5]. Genomic data of Tibetan antelope showed signals of adaptive evolution and expansion of gene families associated with energy metabolism and oxygen transmission [39]. Population analysis of deer mice from different altitudes revealed that Asp64→Gly substitution in α-globin gene is the dominant genotype in the deer mice on the high-altitude localities, which substantially increases oxygen-binding affinity of α-globin [2]. These data show that hypoxia adaptation has genetic foundations and that selection forces favor genetic variations that help to generate more energy molecules under hypoxia.

We compared the coding sequences of PDHE1β between TP and NP locusts and found few genetic variations (Additional file 8); possibly the major difference of this gene lies in the promoter region rather than the coding region. To find the genetic evidence underlying the different hypoxia tolerance of TP and NP locusts, it is necessary to compare their genetic variations on a genome-wide scale, as was done in human using exon capture arrays [5] and SNP arrays [3,4]. Our ongoing locust genome project will help decipher the genomic evidence of hypoxia adaptation in TP locusts in the future. Apart from genetic variations, an increasing number of studies show that epigenetic regulation also plays a crucial role in the cellular response to hypoxia [40-42]. To fully understand the different tolerance of hypoxia between TP and NP locusts, studies in terms of epigenetic regulation of hypoxic responses should be conducted in the future.

## Conclusions

We confirmed the enhanced capacity of TP locusts to tolerate hypoxia, compared their gene expression profiles in field and lab populations, and validated the critical role of aerobic metabolism in TP locusts’ hypoxia tolerance. This study demonstrates that TP and NP locusts have undergone divergence in hypoxia tolerance due to their long-term inhabiting different altitude; it also indicates that insects can adapt to hypoxic pressure by modulating basic metabolic processes. We speculate that genetic variations in basic metabolic processes contribute to their different tolerance of hypoxia, which is worth examining in the future.

## Methods

### Insects

Locusts were collected in the field in 2005 and then raised under experimental conditions as stock cultures. TP locusts were collected in Naidong County, Duilongdeqing County, and Mozhugongka County, whose altitudes were around 4,000 m and *p*O_2_ was around 13 kPa (Figure 1). NP locusts were collected in Wudi County Shandong Province, Huanghua Hebei Province, and Tianjin City, whose altitudes were around 26 m and *p*O_2_ was around 21 kPa (Figure 1). The locusts were taken back to Beijing and raised gregariously as described before [43]. TP locusts have a phenomenon of compulsory diapause and their eggs require about three months in 4°C to terminate diapause, while NP locusts reproduce continuously through the year. We hatched in batches the TP locust eggs belonging to the same generation and there were at most two generations in a year for TP locusts. All the experiments were done with male locusts 10 days after eclosion (Figure 2A).

### Phenotypic assays

Four assays were done to determine the phenotypic differences between NP and TP locusts. (1) Femur length. The left or right femurs were randomly chosen for both male and female locusts and measured using a vernier caliper (2) Stupor ratio. Locusts were subjected to hypoxia in a glass tank placed in a hypoxia chamber; compressed air was diluted with nitrogen to decrease the oxygen content to the required levels. The oxygen level was monitored by an electrode in a hypoxia chamber during the whole process. Five partial oxygen pressures (1.2 kPa, 1.6 kPa, 2.0 kPa, 2.4 kPa and 2.8 kPa) were tested, and six groups of locusts with ten individuals in each group were used at each oxygen level. When the oxygen level was very low, locusts lost coordination, collapsed on the bottom of the glass tank and fell into a motionless state of stupor. Locusts were kept at the test oxygen level for 2 hours and during this period we recorded the number of locusts in stupor every 5 minutes. The number of locusts in stupor fluctuated during the initial minutes of the treatment, but became very stable after 1 hour. We reported the number of locusts in stupor at the end of two-hour treatment. The stupor ratios were calculated at each oxygen levels for TP and NP locusts, and compared using student’s *t*-test after arcsine-square-root transformation. (3) Recovery time. We placed male adult locusts in syringes and then flushed the syringes for five minutes with nitrogen gas so as to clear the atmosphere (Additional file 9). After that, the syringes were sealed tightly and incubated in 30°C for 20 min. Then, the locusts were returned to normoxia, and the time taken by the locusts to become conscious (i.e. stand upright) was recorded as recovery time. Thirty individuals were tested respectively for TP and NP locusts. (4) CO_2_ production. CO_2_ production rates were measured as previously described with minor modifications [10] (Additional files 9 and 10). Five oxygen levels, i.e. 1.6 kPa, 3.2 kPa, 6.5 kPa, 13 kPa and 21 kPa, were selected for comparison. Male locusts subjected to the specified oxygen levels were incubated in a thermostat container for 30 min in 30°C. The locusts were weighed immediately after their CO2 production was measured.

### Microarray experiments and data analysis

Thoracic muscles of field populations were sampled in the field and preserved immediately in RNAlater (Ambion) to prevent RNA degradation. For laboratory populations, after locusts were subjected to 1.6 kPa *p*O_2_ for 2 hours, thoracic muscles were immediately sampled and frozen in the liquid nitrogen. Total RNA was extracted following the protocol of the RNeasy Mini Kit (Qiagen). Then, RNA was reversely transcribed into cDNA, labeled with Cy3 and Cy5, and finally hybridized to locust microarrays as previously described [11-14]. Microarrays were scanned by using a GenePix 4000B microarray scanner and image analysis was performed by using GenePix Pro 6.0 (Axon Instruments). Six independent hybridizations with biological replicates were conducted with a direct-comparison strategy. DEGs were identified with the limma package and the cutoff for DEGs was set as FDR < 0.05 and fold change > 1.5 [11]. DEGs were mapped to gene ontology (GO) terms by using WEGO [44] and to pathways by using KOBAS [45]. All microarray data were MIAME compliant. Both raw and processed data were deposited in the NCBI Gene Expression Omnibus [GEO: GSE43327].

### Real-time quantitative PCR (RT-qPCR)

The standard curve method was used to measure the relative RNA expression level as previously described [11,46]. RT-qPCR amplifications were conducted with an MX3000P spectrofluorometric thermal cycler (Stratagene) and a RealMasterMix (SYBR Green) kit (Tiangen). Melting curve analysis was performed to confirm the specificity of amplification. Primer sequences were presented in Additional file 5.

### Enzyme activities

The enzyme activities of pyruvate dehydrogenase complex (PDH) and NADH dehydrogenase (complex I) in locust flight muscle was measured as previously described [47]. Aconitase activity in locust flight muscle submitochondrial particles was measured by monitoring the increasing rate of cis-aconitase at 240 nm [48]. Briefly, the buffer (50 mM Tris, pH 7.4, 0.6 mM MnCl_2_) was preheated in 30°C for 3 min and then isocitrate (20 mM) was added to the buffer. The mitochondrial protein (final concentration 50 μg · ml^-1^) was then added to the mixture and the absorbance was monitored for 2 min at 30°C to calculate the increasing absorbance rate of cis-aconitate.

### RNAi

Double-stranded RNA (dsRNA) of green fluorescent protein (GFP) and PDHE1β was prepared using Promega T7 RiboMAX™ Express RNAi System following the manufacturer’s instruction (Additional file 5) [12,13]. 18 μg (6 μg/μl) of double-stranded GFP (dsGFP) and dsPDHE1β was injected into male adult locusts at the second ventral segment of the abdomen. Three days later the expression levels of PDHE1α and β were both examined by RT-qPCR to evaluate the interfering efficiency and specificity.

### Measurement of AMP, ADP, and ATP using HPLC

The concentrations of AMP, ADP and ATP were determined using previously established method [49]. Briefly, the samples (i.e., muscle homogenate and isolated mitochondria) were first fixed on ice with a 0.5 volume of 2.3 M perchloric acid for 30 min. The insoluble material was removed via centrifugation, and the supernate was neutralized with 2.5 M KHCO3. Afterward, the supernate was filtered through a 0.45 μm membrane (Agilent, USA) and analyzed via high-performance liquid chromatography (HPLC) at 254 nm using an Agilent 1100 HPLC system (Agilent, USA). Protein concentration was determined via BCA protein assay (Pierce, USA) and used to normalize enzymatic activities and concentrations of AMP, ADP and ATP.

## Abbreviations

NP: North China population; TP: Tibetan population; DEG: Differentially expressed gene; PDH: Pyruvate dehydrogenase; pO2: Partial oxygen pressure.

## Competing interests

The authors declare that they have no competing interests.

## Authors’ contributions

DZ and LK designed the study. DZ, AC and JH designed and performed the respiration experiments. DZ performed the phenotypic assays, microarray experiments, QPCR and RNAi. DZ and ZZ performed measurement of enzymatic activities and HPLC experiments. DZ drafted the manuscript. LK, JH, AC and ZZ revised the manuscript. All authors read and approved the final manuscript.

## Supplementary Material

Additional file 1 **Differentially expressed genes between the field populations of locusts (TP/NP).** The genes were classified into 14 categories according their functions. The possible targets of hypoxia-inducible factor were indicated with asterisk (*). Click here for file

Additional file 2 **Significantly enhanced pathways in field-collected TP locusts.** These pathways were identified using KO-Based Annotation System (KOBAS) with all the genes on the locust microarrays as background. Click here for file

Additional file 3 **Hypoxia-responsive genes in NP locusts.** NP locusts were subjected to 1.6 kPa *p*O_2_ for two hours and then thoracic muscles were sampled. The experiments were done with six biological replicates. Click here for file

Additional file 4 **Hypoxia-responsive genes in TP locusts.** TP locusts were subjected to 1.6 kPa *p*O_2_ for two hours and then thoracic muscles were sampled. The experiments were done with six biological replicates. Click here for file

Additional file 5 **PCR primes used in this study.** Primers used in RT-qPCR and RNAi were listed. Click here for file

Additional file 6 **RT-qPCR validation of microarray results.** Ten genes, including non-differentially expressed genes, were randomly selected for validating the gene expression levels. The fold changes of six biological replicates were averaged and consistent with the microarray results. Click here for file

Additional file 7 **Concentration of AMP, ADP and ATP in locust flight muscle.** The concentrations of AMP, ADP and ATP show no difference between TP and NP locusts under normoxia (A-C); however, they showed difference between TP and NP locusts under extreme hypoxia (D-F) (*p < 0.05, *t*-test, n = 6). Click here for file

Additional file 8 **Multiple sequence alignment of PDHE1β.** The full coding sequence from 15 individuals of TP and NP locusts were sequenced respectively. The nucleotide sequences were translated into peptides using ORF Finder and were aligned with ClustalX. Positions with the same amino acid in all the individuals were labeled with asterisk (*). Click here for file

Additional file 9 **Diagram of respirometry setup.** Gas stream is denoted in thick lines with arrows indicating flow direction. DR, drierite, to remove water vapor; AS, ascarite (Sigma-Aldrich) to scrub CO_2_; FM, flowmeter, to control the flow rate to 120 ml/min; LI-COR, an equipment to quantify CO_2_, connected to UI relaying data to a computer; pump, to pull the air through the respirometry at a constant speed of 100 ml/min; S1, syringe No.1, its plunger is pulled out and then it is plugged with cotton ball; S2, syringe No.2, a locust individual is positioned inside S2 and the air in S2 is evacuated by the gas from the air tank to treat the locust in the specific oxygen; S3, syringe No.3, the respired gas was injected into the respirometry system to quantify the produced CO_2_ using LI-COR. Click here for file

Additional file 10 Description of the respiration experiments. Click here for file
