# Supplementary material for: Efficient utilization of aerobic metabolism helps Tibetan locusts conquer hypoxia
Source: BMC Genomics. 2013 Sep 18;14:631. doi: 10.1186/1471-2164-14-631 (PMC3852963; doi:10.1186/1471-2164-14-631)
Supplement: Additional file 8 — Multiple sequence alignment of PDHE1β. The full coding sequence from 15 individuals of TP and NP locusts were sequenced respectively. The nucleotide sequences were translated into peptides using ORF Finder and were aligned with ClustalX. Positions with the same amino acid in all the individuals were labeled with asterisk (*). [file 1471-2164-14-631-S8.pdf]

```
NP01          MLLPAARFATRRLFSTSAAASAQQMTVRDALNSALDEEMARDDRVFILGEEVALYDGAYK
NP02          MLLPAARFATRRLFSTSAAASAQQMTVRDALNSALDEEMARDDRVFILGEEVALYDGAYK
NP03          MLLPAARFATRRLFSTSAAASAQQMTVRDALNSALDEEMARDDRVFILGEEVALYDGAYK
NP04          MLLPAARFATRRLFSTSAAASAQQMTVRDALNSALDEEMARDDRVFILGEEVALYDGAYK
NP05          MLLPAARFATRRLFSTSAAASAQQMTVRDALNSALDEEMARDDRVFILGEEVALYDGAYK
NP06          MLLPAARFATRRLFSTSAAASAQQMTVRDALNSALDEEMARDDRVFILGEEVALYDGAYK
NP07          MLLPAARFATRRLFSTSAAASAQQMTVRDALNSALDEEMARDDRVFILGEEVALYDGAYK
NP08          MLLPAARFATRRLFSTSAAASAQQMTVRDALNSALDEEMARDDRVFILGEEVALYDGAYK
NP09          MLLPAARFATRRLFSTSAAASAQQMTVRDALNSALDEEMARDDRVFILGEEVALYDGAYK
NP10          MLLPAARFATRRLFSTSAAASAQQMTVRDALNSALDEEMARDDRVFILGEEVALYDGAYK
NP11          MLLPAARFATRRLFSTSAAASAQQMTVRDALNSALDEEMARDDRVFILGEEVALYDGAYK
NP12          MLLPAARFATRRLFSTSAAASAQQMTVRDALNSALDEEMARDDRVFILGEEVALYDGAYK
NP13          MLLPAARFATRRLFSTSAAASAQQMTVRDALNSALDEEMVRDDRVFILGEEVALYDGAYK
NP14          MLLPAARFATRRLFSTSAAASAQQMTVRDALNSALDEEMARDDRVFILGEEVALYDGAYK
NP15          MLLPAARFATRRLFSTSAAASAQQMTVRDALNSALDEEMARDDRVFILGEEVALYDGAYK
TP01          MLLPAARFATRRLFSTSAAASAQQMTVRDALNSALDEEMARDDRVFILGEEVALYDGAYK
TP02          MLLPAARFATRRLFSTSAAASAQQMTVRDALNSALDEEMARDDRVFILGEEVALYDGAYK
TP03          MLLPAARFATRRLFSTSAAASAQQMTVRDALNSALDEEMARDDRVFILGEEVALYDGAYK
TP04          MLLPAARFATRRLFSTSAAASAQQMTVRDALNSALDEEMARDDRVFILGEEVALYDGAYK
TP05          MLLPAARFATRRLFSTSAAASAQQMTVRDALNSALDEEMARDDRVFILGEEVALYDGAYK
TP06          MLLPAARFATRRLFSTSAAASAQQMTVRDALNSALDEEMARDDRVFILGEEVALYDGAYK
TP07          MLLPAARFATRRLFSTSAAASAQQMTVRDALNSALDEEMARDDRVFILGEEVALYDGAYK
TP08          MLLPAARFATRRLFSTSAAASAQQMTVRDALNSALDEEMARDDRVFILGEEVALYDGAYK
TP09          MLLPAARFATRRLFSTSAAASAQQMTVRDALNSALDEEMARDDRVFILGEEVALYDGAYK
TP10          MLLPAARFATRRLFSTSAAASAQQMTVRDALNSALDEEMARDDRVFILGEEVALYDGAYK
TP11          MLLPAARFATRRLFSTSAAASAQQMTVRDALNSALDEEMARDDRVFILGEEVALYDGAYK
TP12          MLLPAARFATRRLFSTSAAASAQQMTVRDALNSALDEEMARDDRVFILGEEVALYDGAYK
TP13          MLLPAARFATRRLFSTSAAASAQQMTVRDALNSALDEEMARDDRVFILGEEVALYDGAYK
TP14          MLLPAARFATRRLFSTSAAASAQQMTVRDALNSALDEEMARDDRVFILGEEVALYDGAYK
TP15          MLLPAARFATRRLFSTSAAASAQQMTVRDALNSALDEEMARDDRVFILGEEVALYDGAYK
              *************************************** ********************

NP01          VSRGLWKKYGDKRVIDTPITEMGFAGLAVGAAMAGLRPICEFMTFNFSMQAIDQVINSAA
NP02          VSRGLWKKYGDKRVIDTPITEMGFAGLAVGAAMAGLRPICEFMTFNFSMQAIDQVINSAA
NP03          VSRGLWKKYGDKRVIDTPITEMGFAGLAVGAAMAGLRPICEFMTFNFSMQAIDQVINSAA
NP04          VSRGLWKKYGDKRVIDTPITEMGFAGLAVGAAMAGLRPICEFMTFNFSMQAIDQVINSAA
NP05          VSRGLWKKYGDKRVIDTPITEMGFAGLAVGAAMAGLRPICEFMTFNFSMQAIDQVINSAA
NP06          VSRGLWKKYGDKRVIDTPITEMGFAGLAVGAAMAGLRPICEFMTFNFSMQAIDQVINSAA
NP07          VSRGLWKKYGDKRVIDTPITEMGFAGLAVGAAMAGLRPICEFMTFNFSMQAIDQVINSAA
NP08          VSRGLWKKYGDKRVIDTPITEMGFAGLAVGAAMAGLRPICEFMTFNFSMQAIDQVINSAA
NP09          VSRGLWKKYGDKRVIDTPITEMGFAGLAVGAAMAGLRPICEFMTFNFSMQAIDQVINSAA
NP10          VSRGLWKKYGDKRVIDTPITEMGFAGLAVGAAMAGLRPICEFMTFNFSMQAIDQVINSAA
NP11          VSRGLWKKYGDKRVIDTPITEMGFAGLAVGAAMAGLRPICEFMTFNFSMQAIDQVINSAA
NP12          VSRGLWKKYGDKRVIDTPITEMGFAGLAVGAAMAGLRPICEFMTFNFSMQAIDQVINSAA
NP13          VSRGLWKKYGDKRVIDTPITEMGFAGLAVGAAMAGLRPICEFMTFNFSMQAIDQVINSAA
NP14          VSRGLWKKYGDKRVIDTPITEMGFAGLAVGAAMAGLRPICEFMTFNFSMQAIDQVINSAA
NP15          VSRGLWKKYGDKRVIDTPITEMGFAGLAVGAAMAGLRPICEFMTFNFSMQAIDQVINSAA
TP01          VSRGLWKKYGDKRVIDTPITEMGFAGLAVGAAMAGLRPICEFMTFNFSMQAIDQVINSAA
TP02          VSRGLWKKYGDKRVIDTPITEMGFAGLAVGAAMAGLRPICEFMTFNFSMQAIDQVINSAA
TP03          VSRGLWKKYGDKRVIDTPITEMGFAGLAVGAAMAGLRPICEFMTFNFSMQAIDQVINSAA
TP04          VSRGLWKKYGDKRVIDTPITEMGFAGLAVGAAMAGLRPICEFMTFNFSMQAIDQVINSAA
TP05          VSRGLWKKYGDKRVIDTPITEMGFAGLAVGAAMAGLRPICEFMTFNFSMQAIDQVINSAA
TP06          VSRGLWKKYGDKRVIDTPITEMGFAGLAVGAAMAGLRPICEFMTFNFSMQAIDQVINSAA
TP07          VSRGLWKKYGDKRVIDTPITEMGFAGLAVGAAMAGLRPICEFMTFNFSMQAIDQVINSAA
TP08          VSRGLWKKYGDKRVIDTPITEMGFAGLAVGAAMAGLRPICEFMTFNFSMQAIDQVINSAA
TP09          VSRGLWKKYGDKRVIDTPITEMGFAGLAVGAAMAGLRPICEFMTFNFSMQAIDQVINSAA
TP10          VSRGLWKKYGDKRVIDTPITEMGFAGLAVGAAMAGLRPICEFMTFNFSMQAIDQVINSAA
TP11          VSRGLWKKYGDKRVIDTPITEMGFAGLAVGAAMAGLRPICEFMTFNFSMQAIDQVINSAA
TP12          VSRGLWKKYGDKRVIDTPITEMGFAGLAVGAAMAGLRPICEFMTFNFSMQAIDQVINSAA
TP13          VSRGLWKKYGDKRVIDTPITEMGFAGLAVGAAMAGLRPICEFMTFNFSMQAIDQVINSAA
TP14          VSRGLWKKYGDKRVIDTPITEMGFAGLAVGAAMAGLRPICEFMTFNFSMQAIDQVINSAA
TP15          VSRGLWKKYGDKRVIDTPITEMGFAGLAVGAAMAGLRPICEFMTFNFSMQAIDQVINSAA
              ************************************************************

NP01          KTFYMSAGSVNVPIVFRGPNGAAAGVAAQHSQCFGAWYSHCPGLKVVSPYSSEDAKGLLK
NP02          KTFYMSAGSVNVPIVFRGPNGAAAGVAAQHSQCFGAWYSHCPGLKVVSPYSSEDAKGLLK
NP03          KTFYMSAGSVNVPIVFRGPNGAAAGVAAQHSQCFGAWYSHCPGLKVVSPYSSEDAKGLLK
NP04          KTFYMSAGSVNVPIVFRGPNGAAAGVAAQHSQCFGAWYSHCPGLKVVSPYSSEDAKGLLK
NP05          KTFYMSAGSVNVPIVFRGPNGAAAGVAAQHSQCFGAWYSHCPGLKVVSPYSSEDAKGLLK
NP06          KTFYMSAGSVNVPIVFRGPNGAAAGVAAQHSQCFGAWYSHCPGLKVVSPYSSEDAKGLLK
NP07          KTFYMSAGSVNVPIVFRGPNGAAAGVAAQHSQCFGAWYSHCPGLKVVSPYSSEDAKGLLK
NP08          KTFYMSAGSVNVPIVFRGPNGAAAGVAAQHSQCFGAWYSHCPGLKVVSPYSSEDAKGLLK
NP09          KTFYMSAGSVNVPIVFRGPNGAAAGVAAQHSQCFGAWYSHCPGLKVVSPYSSEDAKGLLK
NP10          KTFYMSAGSVNVPIVFRGPNGAAAGVAAQHSQCFGAWYSHCPGLKVVSPYSSEDAKGLLK
NP11          KTFYMSAGSVNVPIVFRGPNGAAAGVAAQHSQCFGAWYSHCPGLKVVSPYSSEDAKGLLK
NP12          KTFYMSAGSVNVPIVFRGPNGAAAGVAAQHSQCFGAWYSHCPGLKVVSPYSSEDAKGLLK
```

```
NP13          KTFYMSAGSVNVPIVFRGPNGAAAGVAAQHSQCFGAWYSHCPGLKVVSPYSSEDAKGLLK
NP14          KTFYMSAGSVNVPIVFRGPNGAAAGVAAQHSQCFGAWYSHCPGLKVVSPYSSEDAKGLLK
NP15          KTFYMSAGSVNVPIVFRGPNGAAAGVAAQHSQCFGAWYSHCPGLKVVSPYSSEDAKGLLK
TP01          KTFYMSAGSVNVPIVFRGPNGAAAGVAAQHSQCFGAWYSHCPGLKVVSPYSSEDAKGLLK
TP02          KTFYMSAGSVNVPIVFRGPNGAAAGVAAQHSQCFGAWYSHCPGLKVVSPYSSEDAKGLLK
TP03          KTFYMSAGSVNVPIVFRGPNGAAAGVAAQHSQCFGAWYSHCPGLKVVSPYSSEDAKGLLK
TP04          KTFYMSAGSVNVPIVFRGPNGAAAGVAAQHSQCFGAWYSHCPGLKVVSPYSSEDAKGLLK
TP05          KTFYMSAGSVNVPIVFRGPNGAAAGVAAQHSQCFGAWYSHCPGLKVVSPYSSEDAKGLLK
TP06          KTFYMSAGSVNVPIVFRGPNGAAAGVAAQHSQCFGAWYSHCPGLKVVSPYSSEDAKGLLK
TP07          KTFYMSAGSVNVPIVFRGPNGAAAGVAAQHSQCFGAWYSHCPGLKVVSPYSSEDAKGLLK
TP08          KTFYMSAGSVNVPIVFRGPNGAAAGVAAQHSQCFGAWYSHCPGLKVVSPYSSEDAKGLLK
TP09          KTFYMSAGSVNVPIVFRGPNGAAAGVAAQHSQCFGAWYSHCPGLKVVSPYSSEDAKGLLK
TP10          KTFYMSAGSVNVPIVFRGPNGAAAGVAAQHSQCFGAWYSHCPGLKVVSPYSSEDAKGLLK
TP11          KTFYMSAGSVNVPIVFRGPNGAAAGVAAQHSQCFGAWYSHCPGLKVVSPYSSEDAKGLLK
TP12          KTFYMSAGSVNVPIVFRGPNGAAAGVAAQHSQCFGAWYSHCPGLKVVSPYSSEDAKGLLK
TP13          KTFYMSAGSVNVPIVFRGPNGAAAGVAAQHSQCFGAWYSHCPGLKVVSPYSSEDAKGLLK
TP14          KTFYMSAGSVNVPIVFRGPNGAAAGVAAQHSQCFGAWYSHCPGLKVVSPYSSEDAKGLLK
TP15          KTFYMSAGSVNVPIVFRGPNGAAAGVAAQHSQCFGAWYSHCPGLKVVSPYSSEDAKGLLK
              ************************************************************

NP01          AAIRDPDPVVCLENEILYGVQFPMSDEALSNDFLIPIGKAKIERPGKHVTLVAHSKAVEL
NP02          AAIRDPDPVVCLENEILYGVQFPMSDEALSNDFLIPIGKAKIERPGKHVTLVAHSKAVEL
NP03          AAIRDPDPVVCLENEILYGVQFPMSDEALSNDFLIPIGKAKIERPGKHVTLVAHSKAVEL
NP04          AAIRDPDPVVCLENEILYGVQFPMSDEALSNDFLIPIGKAKIERPGKHVTLVAHSKAVEL
NP05          AAIRDPDPVVCLENEILYGVQFPMSDEALSNDFLIPIGKAKIERPGKHVTLVAHSKAVEL
NP06          AAIRDPDPVVCLENEILYGVQFPMSDEALSNDFLIPIGKAKIERPGKHVTLVAHSKAVEL
NP07          AAIRDPDPVVCLENEILYGVQFPMSDEALSNDFLIPIGKAKIERPGKHVTLVAHSKAVEL
NP08          AAIRDPDPVVCLENEILYGVQFPMSDEALSNDFLIPIGKAKIERPGKHVTLVAHSKAVEL
NP09          AAIRDPDPVVCLENEILYGVQFPMSDEALSNDFLIPIGKAKIERPGKHVTLVAHSKAVEL
NP10          AAIRDPDPVVCLENEILYGVQFPMSDEALSNDFLIPIGKAKIERPGKHVTLVAHSKAVEL
NP11          AAIRDPDPVVCLENEILYGVQFPMSDEALSNDFLIPIGKAKIERPGKHVTLVAHSKAVEL
NP12          AAIRDPDPVVCLENEILYGVQFPMSDEALSNDFLIPIGKAKIERPGKHVTLVAHSKAVEL
NP13          AAIRDPDPVVCLENEILYGVQFPMSDEALSNDFLIPIGKAKIERPGKHVTLVAHSKAVEL
NP14          AAIRDPDPVVCLENEILYGVQFPMSDEALSNDFLIPIGKAKIERPGKHVTLVAHSKAVEL
NP15          AAIRDPDPVVCLENEILYGVQFPMSDEALSNDFLIPIGKAKIERPGKHVTLVAHSKAVEL
TP01          AAIRDPDPVVCLENEILYGVQFPMSDEALSNDFLIPIGKAKIERPGKHVTLVAHSKAVEL
TP02          AAIRDPDPVVCLENEILYGVQFPMSDEALSNDFLIPIGKAKIERPGKHVTLVAHSKAVEL
TP03          AAIRDPDPVVCLENEILYGVQFPMSDEALSNDFLIPIGKAKIERPGKHVTLVAHSKAVEL
TP04          AAIRDPDPVVCLENEILYGVQFPMSDEALSNDFLIPIGKAKIERPGKHVTLVAHSKAVEL
TP05          AAIRDPDPVVCLENEILYGVQFPMSDEALSNDFLIPIGKAKIERPGKHVTLVAHSKAVEL
TP06          AAIRDPDPVVCLENEILYGVQFPMSDEALSNDFLIPIGKAKIERPGKHVTLVAHSKAVEL
TP07          AAIRDPDPVVCLENEILYGVQFPMSDEALSNDFLIPIGKAKIERPGKHVTLVAHSKAVEL
TP08          AAIRDPDPVVCLENEILYGVQFPMSDEALSNDFLIPIGKAKIERPGKHVTLVAHSKAVEL
TP09          AAIRDPDPVVCLENEILYGVQFPMSDEALSNDFLIPIGKAKIERPGKHVTLVAHSKAVEL
TP10          AAIRDPDPVVCLENEILYGVQFPMSDEALSNDFLIPIGKAKIERPGKHVTLVAHSKAVEL
TP11          AAIRDPDPVVCLENEILYGVQFPMSDEALSNDFLIPIGKAKIERPGKHVTLVAHSKAVEL
TP12          AAIRDPDPVVCLENEILYGVQFPMSDEALSNDFLIPIGKAKIERPGKHVTLVAHSKAVEL
TP13          AAIRDPDPVVCLENEILYGVQFPMSDEALSNDFLIPIGKAKIERPGKHVTLVAHSKAVEL
TP14          AAIRDPDPVVCLENEILYGVQFPMSDEALSNDFLIPIGKAKIERPGKHVTLVAHSKAVEL
TP15          AAIRDPDPVVCLENEILYGVQFPMSDEALSNDFLIPIGKAKIERPGKHVTLVAHSKAVEL
              ************************************************************

NP01          CLNAASQLAGEGIEAEVINLRSLRPLDEEAIVKSVVKTNHLITVEQGWPQCGIGAEISAR
NP02          CLNAASQLAGEGIEAEVINLRSLRPLDEEAIVKSVVKTNHLITVEQGWPQCGIGAEISAR
NP03          CLNAASQLAGEGIEAEVINLRSLRPLDEEAIVKSVVKTNHLITVEQGWPQCGIGAEISAR
NP04          CLNAASQLAGEGIEAEVINLRSLRPLDEEAIVKSVVKTNHLITVEQGWPQCGIGAEISAR
NP05          CLNAASQLAGEGIEAEVINLRSLRPLDEEAIVKSVVKTNHLITVEQGWPQCGIGAEISAR
NP06          CLNAASQLAGEGIEAEVINLRSLRPLDEEAIVKSVVKTNHLITVEQGWPQCGIGAEISAR
NP07          CLNAASQLAGEGIEAEVINLRSLRPLDEEAIVKSVVKTNHLITVEQGWPQCGIGAEISAR
NP08          CLNAASQLAGEGIEAEVINLRSLRPLDEEAIVKSVVKTNHLITVEQGWPQCGIGAEISAR
NP09          CLNAASQLAGEGIEAEVINLRSLRPLDEEAIVKSVVKTNHLITVEQGWPQCGIGAEISAR
NP10          CLNAASQLAGEGIEAEVINLRSLRPLDEEAIVKSVVKTNHLITVEQGWPQCGIGAEISAR
NP11          CLNAASQLAGEGIEAEVINLRSLRPLDEEAIVKSVVKTNHLITVEQGWPQCGIGAEISAR
NP12          CLNAASQLAGEGIEAEVINLRSLRPLDEEAIVKSVVKTNHLITVEQGWPQCGIGAEISAR
NP13          CLNAASQLAGEGIEAEVINLRSLRPLDEEAIVKSVVKTNHLITVEQGWPQCGIGAEISAR
NP14          CLNAASQLAGEGIEAEVINLRSLRPLDEEAIVKSVVKTNHLITVEQGWPQCGIGAEISAR
NP15          CLNAASQLAGEGIEAEVINLRSLRPLDEEAIVKSVVKTNHLITVEQGWPQCGIGAEISAR
TP01          CLNAASQLAGEGIEAEVINLRSLRPLDEEAIVKSVVKTNHLITVEQGWPQCGIGAEISAR
TP02          CLNAASQLAGEGIEAEVINLRSLRPLDEEAIVKSVVKTNHLITVEQGWPQCGIGAEISAR
TP03          CLNAASQLAGEGIEAEVINLRSLRPLDEEAIVKSVVKTNHLITVEQGWPQCGIGAEISAR
TP04          CLNAASQLAGEGIEAEVINLRSLRPLDEEAIVKSVVKTNHLITVEQGWPQCGIGAEISAR
TP05          CLNAASQLAGEGIEAEVINLRSLRPLDEEAIVKSVVKTNHLITVEQGWPQCGIGAEISAR
TP06          CLNAASQLAGEGIEAEVINLRSLRPLDEEAIVKSVVKTNHLITVEQGWPQCGIGAEISAR
TP07          CLNAASQLAGEGIEAEVINLRSLRPLDEEAIVKSVVKTNHLITVEQGWPQCGIGAEISAR
TP08          CLNAASQLAGEGIEAEVINLRSLRPLDEEAIVKSVVKTNHLITVEQGWPQCGIGAEISAR
TP09          CLNAASQLAGEGIEAEVINLRSLRPLDEEAIVKSVVKTNHLITVEQGWPQCGIGAEISAR
```

```
TP10            CLNAASQLAGEGIEAEVINLRSLRPLDEEAIVKSVVKTNHLITVEQGWPQCGIGAEISAR
TP11            CLNAASQLAGEGIEAEVINLRSLRPLDEEAIVKSVVKTNHLITVEQGWPQCGIGAEISAR
TP12            CLNAASQLAGEGIEAEVINLRSLRPLDEEAIVKSVVKTNHLITVEQGWPQCGIGAEISAR
TP13            CLNAASQLAGEGIEAEVINLRSLRPLDEEAIVKSVVKTNHLITVEQGWPQCGIGAEISAR
TP14            CLNAASQLAGEGIEAEVINLRSLRPLDEEAIVKSVVKTNHLITVEQGWPQCGIGAEISAR
TP15            CLNAASQLAGEGIEAEVINLRSLRPLDEEAIVKSVVKTNHLITVEQGWPQCGIGAEISAR
                ************************************************************

NP01            IMESEAFYHLDSPVIRVTGVDTPMPYCKTLEENALPQLKDVVEAVKKVLGVK
NP02            IMESEAFYHLDSPVIRVTGVDTPMPYCKTLEENALPQLKDVVEAVKKVLGVK
NP03            IMESEAFYHLDSPVIRVTGVDTPMPYCKTLEENALPQLKDVVEAVKKVLGVK
NP04            IMESEAFYHLDSPVIRVTGVDTPMPYCKTLEENALPQLKDVVEAVKKVLGVK
NP05            IMESEAFYHLDSPVIRVTGVDTPMPYCKTLEENALPQLKDVVEAVKKVLGVK
NP06            IMESEAFYHLDSPVIRVTGVDTPMPYCKTLEENALPQLKDVVEAVKKVLGVK
NP07            IMESEAFYHLDSPVIRVTGVDTPMPYCKTLEENALPQLKDVVEAVKKVLGVK
NP08            IMESEAFYHLDSPVIRVTGVDTPMPYCKTLEENALPQLKDVVEAVKKVLGVK
NP09            IMESEAFYHLDSPVIRVTGVDTPMPYCKTLEENALPQLKDVVEAVKKVLGVK
NP10            IMESEAFYHLDSPVIRVTGVDTPMPYCKTLEENALPQLKDVVEAVKKVLGVK
NP11            IMESEAFYHLDSPVIRVTGVDTPMPYCKTLEENALPQLKDVVEAVKKVLGVK
NP12            IMESEAFYHLDSPVIRVTGVDTPMPYCKTLEENALPQLKDVVEAVKKVLGVK
NP13            IMESEAFYHLDSPVIRVTGVDTPMPYCKTLEENALPQLKDVVEAVKKVLGVK
NP14            IMESEAFYHLDSPVIRVTGVDTPMPYCKTLEENALPQLKDVVEAVKKVLGVK
NP15            IMESEAFYHLDSPVIRVTGVDTPMPYCKTLEENALPQLKDVVEAVKKVLGVK
TP01            IMESEAFYHLDSPVIRVTGVDTPMPYCKTLEENALPQLKDVVEAVKKVLGVK
TP02            IMESEAFYHLDSPVIRVTGVDTPMPYCKTLEENALPQLKDVVEAVKKVLGVK
TP03            IMESEAFYHLDSPVIRVTGVDTPMPYCKTLEENALPQLKDVVEAVKKVLGVK
TP04            IMESEAFYHLDSPVIRVTGVDTPMPYCKTLEENALPQLKDVVEAVKKVLGVK
TP05            IMESEAFYHLDSPVIRVTGVDTPMPYCKTLEENALPQLKDVVEAVKKVLGVK
TP06            IMESEAFYHLDSPVIRVTGVDTPMPYCKTLEENALPQLKDVVEAVKKVLGVK
TP07            IMESEAFYHLDSPVIRVTGVDTPMPYCKTLEENALPQLKDVVEAVKKVLGVK
TP08            IMESEAFYHLDSPVIRVTGVDTPMPYCKTLEENALPQLKDVVEAVKKVLGVK
TP09            IMESEAFYHLDSPVIRVTGVDTPMPYCKTLEENALPQLKDVVEAVKKVLGVK
TP10            IMESEAFYHLDSPVIRVTGVDTPMPYCKTLEENALPQLKDVVEAVKKVLGVK
TP11            IMESEAFYHLDSPVIRVTGVDTPMPYCKTLEENALPQLKDVVEAVKKVLSVT
TP12            IMESEAFYHLDSPVIRVTGVDTPMPYCKTLEENALPQLKDVVEAVKKVLGVK
TP13            IMESEAFYHLDSPVIRVTGVDTPMPYCKTLEENALPQLKDVVEAVKKVLGVK
TP14            IMESEAFYHLDSPVIRVTGVDTPMPYCKTLEENALPQLKDVVEAVKKVLGVK
TP15            IMESEAFYHLDSPVIRVTGVDTPMPYCKTLEENALPQLKDVVEAVKKVLGVK
                ************************************************* *
```
