# Supplementary material for: Efficient utilization of aerobic metabolism helps Tibetan locusts conquer hypoxia
Source: BMC Genomics. 2013 Sep 18;14:631. doi: 10.1186/1471-2164-14-631 (PMC3852963; doi:10.1186/1471-2164-14-631)
Supplement: Additional file 10 — Description of the respiration experiments. [file 1471-2164-14-631-S10.docx]

**Supplementary text describing the respiration experiments**

Respiration was measured through a constant volume technique with the LI-6252 CO_2_ analyzer (LI-COR Inc., USA) ([Greenlee and Harrison 2004](#_ENREF_1)). The system used compressed air tanks containing different fractions of oxygen balanced with nitrogen to flush the system (Figure S4). The air stream flowed from the tanks through a column containing magnesium perchlorate (Fisher Scientific) and ascarite (Sigma-Aldrich) to remove any remaining water vapor and CO_2_, and then a gas flow meter (OMEGA FMA-5606) was used to control the air flow at 120ml/min. A three-way connector was used to split the air stream into two branches, one for flushing the syringes containing locusts (Figure S4, Syr2) and the other for measuring the CO_2_ production after treatments (Figure S4, Syr1). Dry, CO2-free air was pulled sequentially through a length of tubing, a drying column, and the LICOR CO2 analyzer by a downstream pump. Respiration rates were measured for locusts over 30 min. For measurement of CO2 emission rate by the locusts, the plunger of the syring containing the locust was depressed, injecting 25 ml into the air stream entering the CO2 analyzer. The subsequent peak in CO2 was integrated and converted to moles using a Sable Systems UI2 interface and Sable Systems ExpeData data acquisition and analysis software.

**Reference:**

Greenlee, K. J. and J. F. Harrison (2004). "Development of respiratory function in the American locust *Schistocerca americana* I. Across-instar effects." Journal of Experimental Biology **207**(3): 497-508.
